# *Balticalcarus archibaldi* Simutnik Gen. et sp. n. (*Chalcidoidea*, *Encyrtidae*) with the Unusually Small Mesotibial Spur from Baltic Amber

**DOI:** 10.3390/life12122028

**Published:** 2022-12-05

**Authors:** Serguei A. Simutnik, Evgeny E. Perkovsky, Dmitry V. Vasilenko

**Affiliations:** 1 I.I. Schmalhausen Institute of Zoology, National Academy of Sciences of Ukraine, 01030 Kiev, Ukraine; 2 A.A. Borissiak Paleontological Institute, Russian Academy of Sciences, Moscow 117647, Russia; 3 Paleontological Laboratory, Cherepovets State University, Cherepovets 162600, Russia

**Keywords:** evolution of Encyrtidae, Tetracnemini, common ancestor, hypopygium

## Abstract

*Balticalcarus archibaldi* Simutnik, gen. et sp. n., is described and illustrated based on a female specimen from late Eocene Baltic amber. The new genus is characterized by the absence of a filum spinosum, a “boat”-shaped hypopygium enclosing the ovipositor, reaching far past the apex of the syntergum, the presence of a line of long setae along the entire costal cell of the hind wing, and a transverse line of thickened setae alongside the hyaline spur vein. Moreover, like most previously described Eocene Encyrtidae, the new taxon differs from the majority of the extant ones by a number of morphological features. The new fossil differs from most extant and all known fossil Encyrtidae by its unusually small, thin, smooth (without microsetae) mesotibial spur.

## 1. Introduction

To date, 17 species in 15 extinct genera of Encyrtidae are described from the Rovno, Baltic, and Danish ambers. *Glaesus gibsoni* Simutnik, 2014 and *Eocencnemus gedanicus* Simutnik, 2014 have been described based on male specimens from late Eocene Baltic amber [1] and several undescribed encyrtids have been reported by Noyes and Hayat [2] and Manukyan [3]. Females of *Eocencyrtus zerovae* Simutnik, 2001 (Encyrtidae) and another Chalcidoidea with a large and setose mesotibial spur, *Leptoomus janzeni* Gibson, 2008, were recorded from both Baltic and Rovno ambers [1,4]. *Sulia glaesaria* Simutnik, 2015 (Encyrtidae), originally described from late Eocene Danish amber, was then reported in coeval Rovno amber [5,6]. The previously studied Encyrtidae from late Eocene European ambers differ from most extant species by a number of morphological features [1,5,6,7,8,9,10,11,12].

One species of the extant genus *Copidosoma* Ratzeburg, 1844, *C. archeodominica* Zuparko and Trjapitzin, 2014, was described from Miocene Dominican amber [13].

The earliest known Encyrtidae were described from middle Eocene Sakhalinian amber [7,12,14,15]. All of these are characterized by their cerci located at the gastral apex and possession of a long, thick, and setose mesotibial spur. A new fossil with an unusually small, bare (without microsetae) mesotibial spur and cerci advanced is described here.

## 2. Materials and Methods

High precipitation and mild winters set the conditions for the thriving mixed mesophytic conifer–angiosperm Baltic amber forest [16], which had a mixture of tropical and “Holarctic” biotic elements very unusual in the modern world [17,18,19], where “Holarctic” ones strongly dominate [16,18,19,20,21,22,23,24].

The studied specimen is part of the unbiased PIN-964 Baltic amber collection of the Borissiak Paleontological Institute of the Russian Academy of Sciences, Moscow (PIN). This material was collected in 1948 by A.G. Sharov directly in the amber processing factory in the Yantarnyi settlement, Kaliningrad Oblast [25].

The specimen was examined using the equipment and techniques described in Simutnik et al. [9]. Photographs were taken using a Leica Z16 APO stereomicroscope equipped with a Leica DFC 450 camera and processed with LAS Core and Adobe Photoshop software (brightness and contrast only).

The terminology and abbreviations follow Sharkov [26], Gibson [27], and Heraty et al. [28]. We use the following abbreviations: **F1, F2, etc.** = funicular segments 1, 2, etc.; **LOL** = minimum distance between the anterior ocellus and a posterior ocellus; **OOL** = minimum distance between an eye margin and the adjacent posterior ocellus; **OCL** = minimum distance between a posterior ocellus and the occipital margin; **POL** = minimum distance between the posterior ocelli.

## 3. Results

### Systematic Paleontology

**Chalcidoidea Latreille, 1817**

**Encyrtidae Walker, 1837**

**Tetracneminae Howard, 1892**

**Genus *Balticalcarus* Simutnik gen. nov.**

**Figure 1:**
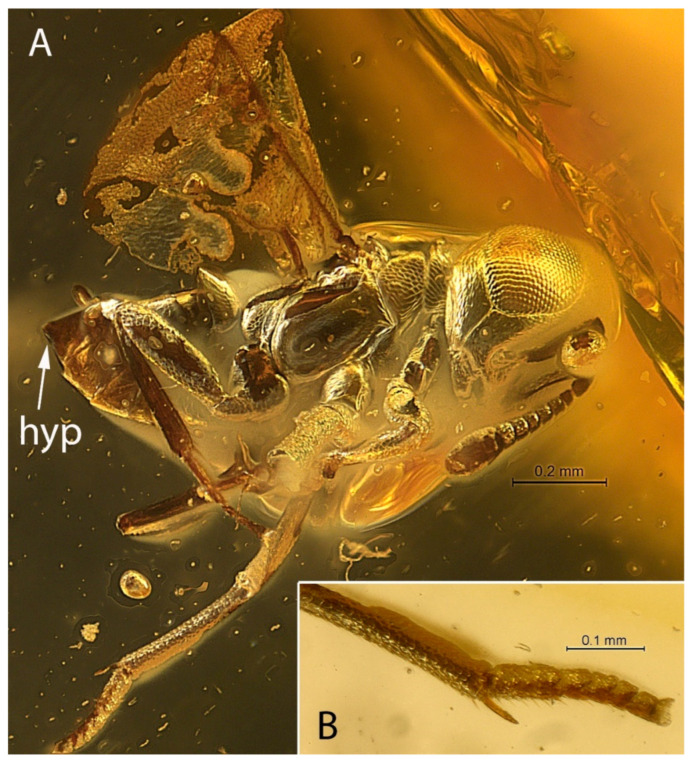
*Balticalcarus archibaldi* gen. et sp. nov., holotype female (**A**) body, lateral (hyp—hypopygium) (**B**) mesotibia with spur and tarsus. Scale bars: 0.2 mm (**A**), 0.1 mm (**B**).

**Figure 2:**
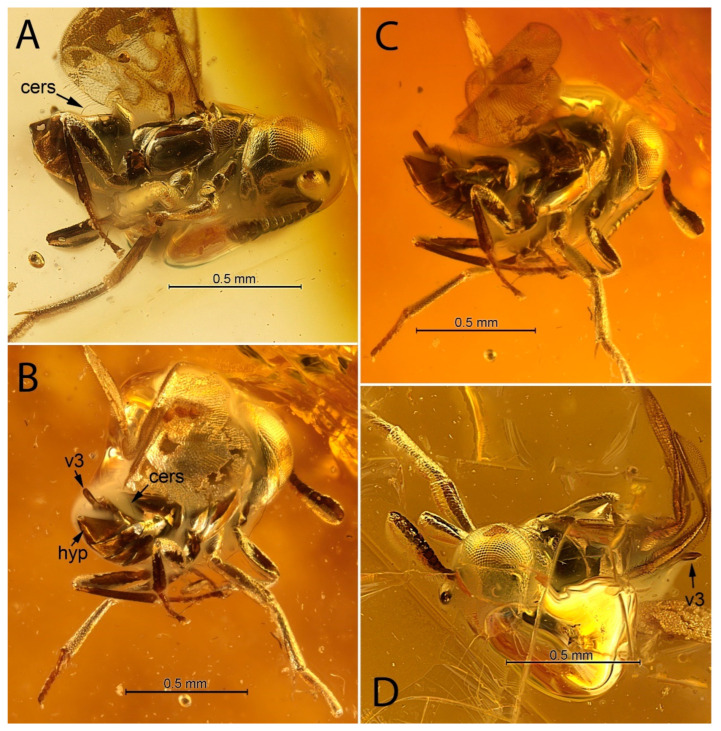
*Balticalcarus archibaldi* gen. et sp. nov., holotype female, body (**A**) lateral (cers—cercal seta) (**B**), posterolateral (hyp—hypopygium, v3—ovipositor sheaths), (**C**) posterolateral (**D**) anteriodorsal. Scale bars: 0.5 mm.

**Figure 3:**
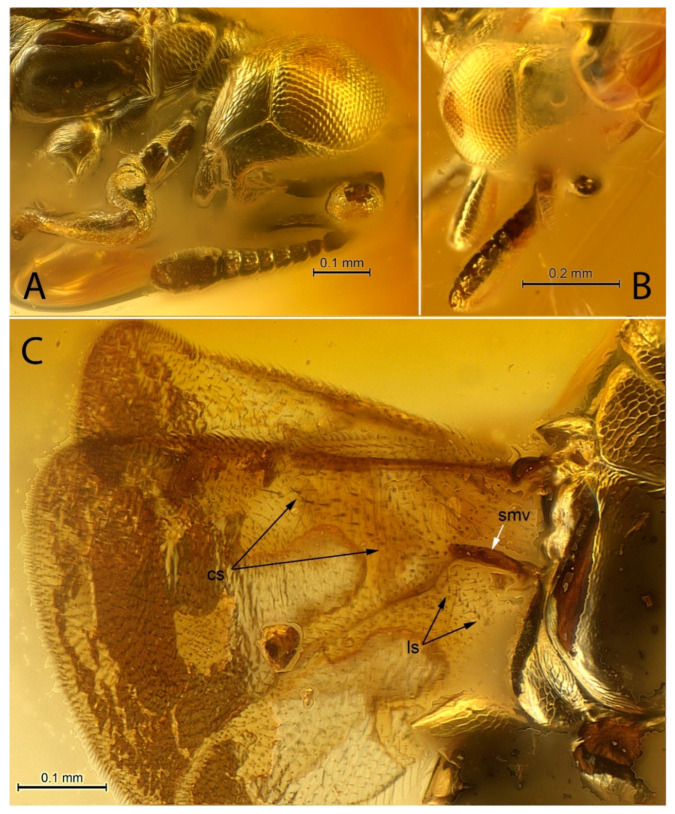
*Balticalcarus archibaldi* gen. et sp. nov., holotype female (**A**) head, mandible, antenna, part of mesosoma, ventrolateral, (**B**) head, antenna, anterodorsal, (**C**) wings, part of mesosoma, dorsolateral (cs—covering setae, ls—transverse line of thickened setae alongside and basal to hyaline spur vein, smv—swollen part of submarginal vein)). Scale bars: 0.1 mm (**A**,**C**), 0.2 mm (**B**).

**Figure 4:**
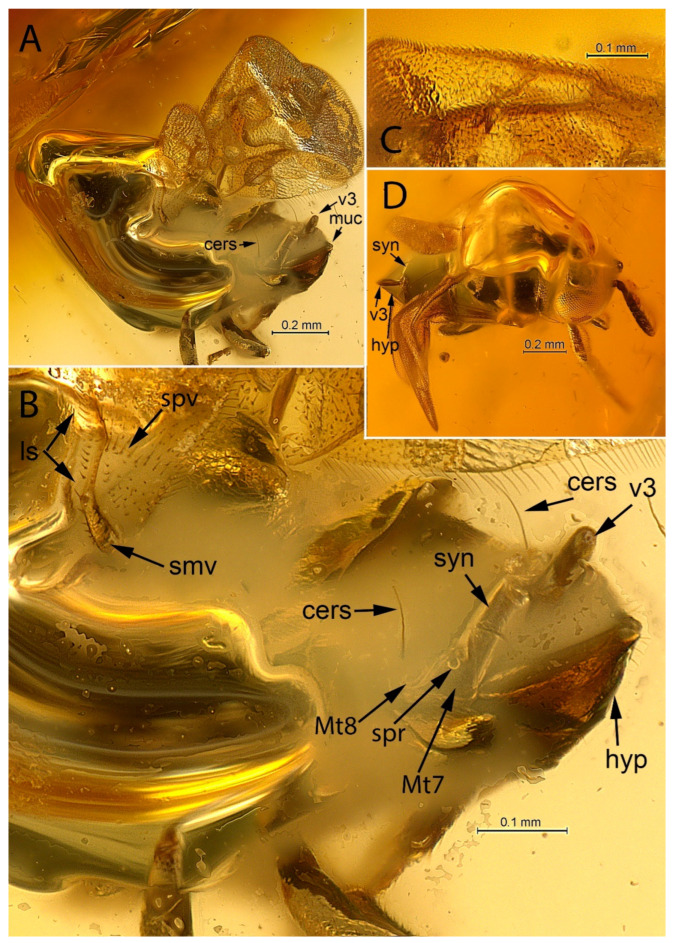
*Balticalcarus archibaldi* gen. et sp. nov., holotype female (**A**) body, posterolateral (cers—cercal seta, muc—mucro, v3—ovipositor sheaths), (**B**) gaster, posterolateral (hyp—hypopygium, ls—line of setae, smv—submarginal vein, spr—spiracle on the lateral lobe of the Mt7, spv—hyaline spur vein, syn—syntergum), (**C**) venation of forewing, (**D**) body, dorsal. Scale bars: 0.2 mm (**A**,**D**), 0.1 mm (**B**,**C**).

Figure 1, Figure 2, Figure 3 and Figure 4.

urn:lsid:zoobank.org:act:FEAEBD6F-98B2-46DB-8A38-D09F3F58A823

(accessed on 02.12.2022)

**Type species.***Balticalcarus archibaldi* Simutnik, **sp. nov.**

**Species composition.** Type species only.

**Etymology.** The name of the genus is a combination of the words “Baltic” and “calcar”. The new genus is distinguished by an unusual mesotibial spur (Latin: *calcar* = spur). The genus name is a masculine noun.

**Diagnosis. Female.** Body compact, not flattened, with large, hypognathous head and large eyes; F1 shorter than broad; mandible, probably 2-dentate (Figure 3A); filum spinosum absent; covering setae present; postmarginal vein longer than marginal vein; costal cell of hind wing with line of long setae, the longest of which is located alongside parastigma (Figure 4B: ls); row of thickened setae present alongside hyaline spur vein of hind wing (Figure 3C: ls; Figure 4B); mesotibia almost without extension to apex; mesotibial spur very small, thin, bare, slightly curved inwards; mesobasitarsus relatively short (Figure 1B); cerci located in apical third of metasoma; hypopygium “boat”-shaped and enclosing the ovipositor, its apex reaching far past apex of last gastral tergum (Figure 4D).

**Male**. Unknown.

**Remarks.** Placement of the *Balticalcarus archibaldi* gen. et sp. nov. in Tetracneminae is supported by the absence of the filum spinosum of the linea calva, its bidentate mandibles, and the hypopygium reaching far past the apex of the syntergum. However, the connection of Mt8 and the outer plates of the ovipositor by the paratergites (the presence of which is one of the main features of Tetracneminae, see Trjapitzin [29]) are not distinctly visible in the type specimen. The structure labeled Mt8 and indicated by an arrow in Figure 4B might be the paratergite running anteriorly to the outside of the cercal plate.

Such a small mesotibial spur has never been recorded before in fossil encyrtids and is rare among extant ones (e.g., in *Trjapitzinellus* Viggiani, 1967; *Platyrhopus* Erdös, 1955 (Encyrtinae); and some genera of Miraini Ashmead, 1900 sensu Trjapitzin [29] (Tetracneminae)). However, the mesotibial spur of these extant genera is usually straight, thick, and densely covered with microsetae; the apex of the mesotibia is also considerably thickened and the basitarsus elongated.

The hind wing of the new genus has a single line of long setae along the entire costal cell (Figure 4B: ls) as in most extant Tanaostigmatidae, extinct *Leptoomus janzeni* (Figure 2E in [4]), and some extant genera of Bothriothoracini Howard, 1895 (Encyrtinae) [8,30]). The longest of these setae are located along the parastigma. In fossil Encyrtidae, the same line of long setae has been recorded in late Eocene *Eocencnemus sugonjaevi* Simutnik, 2002, *Sulia glaesaria* [8], and *Electronoyesella* [11], which do not belong to Encyrtinae. A line of long, but sparser and more or less equal in length setae along the costal cell of the hind wing is also present in the earliest known, middle Eocene encyrtids from Sakhalinian amber and the extant genus *Ericydnus* Walker, 1837 [11].

A transverse row of thickened setae alongside the spur vein of the hind wing (Figure 3C: ls, Figure 4B: spv) has been also found in late Eocene *Electronoyesella* only [11]. It is absent in all known extant encyrtids, tanaostigmatids, late Eocene *Leptoomus,* and *Eocencnemus, Sulia*, as well as in all middle Eocene encyrtids from Sakhalinian amber.

***Balticalcarus archibaldi* Simutnik, sp. nov.**

urn:lsid:zoobank.org:act:1E969BFB-AEDF-4089-AD48-EC12F6C6A027

(accessed on 02.12.2022)

Figure 1, Figure 2, Figure 3 and Figure 4.

**Material. *Holotype***, PIN 964/1097, 1 ♀, Yantarnyi; Baltic amber; late Eocene. The inclusion is in a yellow and clear piece of amber (ca. 11 × 9 × 4 mm). The specimen is well preserved, but its wings are deformed and its left side is obscured by a large air bubble (Figure 2D and Figure 4A).

**Syninclusions.** None.

**Etymology.** Named in honor of paleoentomologist S. Bruce Archibald.

**Description. Female.** Habitus as in Figure 1A and Figure 2. Body length 1.3 mm.

***Coloration***. Body black-brown; antenna unicolorous, dark brown; venation brown; mesotibial spur and tarsi pale yellowish-brown; surface of frontovertex, thorax (part), and legs appear shiny due to a thin layer of air, but without visible metallic shine.

***Sculpture.*** Head, pronotum, mesoscutum, scutellum, and prepectus rough reticulate; scape and pedicel, tegula, coxae, and legs, also relatively similar reticulate; mesopleuron and gaster with smoother sculpturing.

***Head.*** Lenticular, slightly wider than thorax in dorsal view (Figure 2B,D and Figure 4D), broader and then long; occipital margin sharp, but not carinate, with row of short black setae (Figure 3B); eyes bare, without visible setae (Figure 2A–D), inner orbits parallel; frontovertex slightly longer than broad, minimum distance between eyes about 0.37× head width; ocelli forming a slightly <90° angle; anterior ocellus closer to upper margin of scrobal depression than to occipital margin; posterior ocelli elliptical in dorsal view, located closer to eye margin than to occipital margin; OOL about 0.5× ocellar diameter; OOL:POL:LOL:OCL about 1:10:7:3; eye reaching occipital margin (Figure 2B); antennal scrobes as in Figure 3A,B, poorly visible, but meeting dorsally, not extended to anterior ocellus, in dorsal view anterior ocellus approximately three times closer to upper margin of scrobal depression than to occipital margin; interantennal prominence as in Figure 3A; antennal toruli located closer to mouth margin than to level of lower margin of eyes, separated from mouth margin by distance slightly less than their own width (Figure 3A); malar space with complete malar sulcus, about 0.3× height of eye.

***Antenna*.** Geniculate, with six funicular segments and three-segmented clava; radicle short, about 1.5× as long as broad (Figure 3A); antennal scape including radicle ~7× as long as broad, flattened, reticulate; pedicel conical, about as long as first two funicular segments combined, longer than any funicle segment; F1 slightly shorter than broad, F2 and F3 subquadrate, F4–F6 distinctly broader than long; width of flagellomeres slightly increases toward apex; at least F2–F6, and basal segment of clava with mps; clava as long as F3–F6 combined, without oblique truncation (Figure 3A,B), flattened, much wider than F6; flagellum and clava clothed in short setae.

***Mesosoma*.** Pronotum short; notauli and meeting of axillae not visible in holotype; scutellum slightly convex (Figure 3B), apically pointed (Figure 3C); prepectus large; mesopleuron long, enlarged posteriorly; metapleuron triangular, narrow, without visible setation (Figure 3C); propodeum bare, with relatively large lateral parts, touching hind coxa (Figure 3C).

***Wings*.** Fully developed, hyaline; linea calva closed ventrally, with well-developed line of long setae alongside its basal margin (Figure 3C: cs); parastigma thickened (Figure 4C), hyaline break (unpigmented area) present; marginal vein about 5× as long as broad; stigmal vein as long as marginal, with long uncus (Figure 4C); postmarginal vein almost 2× as long as marginal vein, enlarged seta marking apex of postmarginal vein absent (as long as others on this vein); setae of marginal fringe short; hind wing with basal part of submarginal vein strongly swollen (Figure 3B and Figure 4C: smv).

***Legs*.** Apex of mid tibia not expanded, with at least one apical peg along lateroapical edge (Figure 1B); mesotibial spur thin, slightly curved, bare, about 0.5× basal mesotarsal segment, relatively short, as long as 2–4 tarsal segments combined; ventral surface of mesobasitarsus with differentiated setation; tarsi five-segmented.

***Metasoma*.** As long as mesosoma; cerci situated in apical third of metasoma, with long vertical, curved setae (Figure 2A,B and Figure 4B: cers); syntergum (Mt8 + Mt9) v-shaped, no longer than 1/3 of metasoma; possible paratergite running anteriorly to outside of cercal plate is arrowed as Mt8 in Figure 4B; apex of hypopygium with mucro, reaching far beyond apex of syntergum (Figure 1A and Figure 4B,D); lateral margin of hypopygium bare, without row of setae; gonostyli with extending parts as long as mesobasitarsus (Figure 2B,C and Figure 4A,B,D: v3).

**Male.** Unknown.

Genus composition. Type species only.

## 4. Discussion

According to the modern molecular and intricate combined analyses of Munro et al. [31] and Cruad et al. [32] (and references therein), the evolutionary history of Encyrtidae began over 100 million years ago during the Cretaceous, when Chalcidoidea underwent a rapid radiation. Along with several other families of “soft bodied” chalcidoids of the “Tiny Wasp clade”, Encyrtidae diverged soon after. The first lineages to diverge (Mymaridae, Baeomorphidae (Rotoitidae), and “Tiny Wasp clade”) were likely first oophagous and later associated mostly with hemipteran hosts [32].

According to all molecular analyses, both subfamilies of Encyrtidae (Encyrtinae and Tetracneminae) were in existence by the second half of the Cretaceous.

There are reliable reports of some Chalcidoidea from the Cretaceous ambers [33,34,35,36,37], but despite active searches, Encyrtidae are still unknown. Their earliest fossils are from Sakhalinian amber. Kodrul [38] convincingly dated the Naibuchi Formation in which Sakhalinian amber is found in situ as the middle Eocene (43–47 Ma) based on geological and paleobotanical data and Baltic, Rovno and Danish ambers are estimated to be the late Eocene (34–38 Ma) [18,19,20,21,22,23,24,25,39,40]. The comparative morphological analysis of middle and late Eocene encyrtid fossils further support Sakhalinian amber being older than European ambers [7,12]. The phylogenetic relationships of Sakhalinian and extant encyrtids at the subfamily level remain unresolved. Sakhalinian encyrtids differ from both extant and late Eocene European amber encyrtids by a number features [12], e.g., their cerci are located close to each other, extremely close to the apex of the gaster.

The earliest reliable morphological evidence for the existence of both extant encyrtid subfamilies were in the late Eocene [1,11]. The filum spinosum is the short and thickened setae on the apical margin of the linea calva that function as a part of the wing-coupling mechanism at the moment of jumping and takeoff. This is one of the main features of the extant Encyrtinae: Trjapitzin [41]. The filum spinosum was only reported since the late Eocene, not in middle Eocene Sakhalinian amber [12]. The oldest known encyrtine is a late Eocene fossil of the genus *Glaesus* Simutnik, 2014 in Baltic amber, and then several other genera with the filum spinosum were reported in Danish and Rovno ambers.

The presence of paratergites between the syntergum and the outer plates of the ovipositor is one of the main features of Tetracneminae [41]. We have only recently found this sclerotized, ribbon-like structure in a Rovno amber encyrtid wasp for the first time [11] (Figure 7). However, there are several taxa lacking the filum spinosum, and paratergites are unknown among them. Therefore, it would be premature to classify them as members of the Tetracneminae and their taxonomic placement within the family remains uncertain.

A reliable fossil of the extant genus is recorded in the Miocene [13]. The phylogenetic relationships of late Eocene encyrtids to extant genera and tribes remain unresolved. Most described Eocene Encyrtidae differ from the majority of extant ones by their long forewing veins including the marginal vein, a distinctly thickened, but not triangular parastigma, a seta marking the apex of the postmarginal vein is not any longer than others on this vein, and a very short radicle. They have poorly differentiated sculpture and are always fully winged, which are without distinct infusions, stripes, or patterns. Almost all retain the apical or subapical positions of their cerci. Cerci that are extremely advanced to the base of the metasoma, as in many extant members, are unknown in Eocene Encyrtidae.

Almost certainly *Balticalcarus* also possess paratergites (see Mt8 in Figure 4B) and belong to Tetracneminae. According to J.S. Noyes [30], the new taxon is probably very close to the common ancestor of the group of genera near the extant *Clausenia* Ishii, 1923, *Mohelencyrtus* Hoffer, 1969, and maybe the whole lineage that includes *Charitopus* Förster, 1856. Its “boat-shaped” hypopygium that encloses the ovipositor is very reminiscent of *Charitopus*, *Lyka* Mercet, 1921, etc., and the forewing venation is very similar to that of *Clausenia* and *Moraviella* Hoffer, 1954, and perhaps *Mohelencyrtus*. Its short mesotibial spur is also characteristic of this group. Apparently, all of these genera (including those of the tribe Miraini sensu Trjapitzin [29]) could be placed in the Tetracnemini Howard, 1892, because their ovipositor structures are so characteristic of the group [30]. However, the short antenna of *Balticalcarus* is not typical of this group and the mesotibial spur of all of these extant genera is usually straight, thick, and densely covered with microsetae. In any case, the discovery of this fossil is the next small step towards understanding the evolution of encyrtids.
